# Achromatic Non-Interferometric Single Grating Neutron Dark-Field Imaging

**DOI:** 10.1038/s41598-019-55558-0

**Published:** 2019-12-23

**Authors:** M. Strobl, J. Valsecchi, R. P. Harti, P. Trtik, A. Kaestner, C. Gruenzweig, E. Polatidis, J. Capek

**Affiliations:** 1 Laboratory for Neutron Scattering and Imaging, Paul Scherrer Institut, 5232 Villigen, Switzerland; 2 Niels Bohr Institute, University of Copenhagen, Nørregade 10, 1165 Copenhagen, Denmark

**Keywords:** Structure of solids and liquids, Imaging techniques

## Abstract

We demonstrate a simple single grating beam modulation technique, which enables the use of a highly intense neutron beam for differential phase and dark-field contrast imaging and thus spatially resolved structural correlation measurements in full analogy to interferometric methods. In contrast to these interferometric approaches our method is intrinsically achromatic and provides unprecedented flexibility in the choice of experimental parameters. In particular the method enables straight forward application of quantitative dark-field contrast imaging in time-of-flight mode at pulsed neutron sources. Utilizing merely a macroscopic absorption mask unparalleled length scales become accessible. We present results of quantitative dark-field contrast imaging combining microstructural small angle scattering analyses with real space imaging for a variety of materials.

## Introduction

Neutron interferometry has created significant impact in neutron imaging in particular when Talbot Lau grating interferometers enabled unprecedented high efficiency phase contrast imaging with neutrons early in the millennium^1^. Attempts to utilize interferometry and its outstanding sensitivity to quantum beam phase effects for spatially resolved measurements have been reported earlier and in particular with single crystal Mach-Zehnder interferometers^2^. However, their coherence and stability requirements as well as spatial restrictions hindered practical applications. Also attempted non-interferometric techniques suffered from high coherence requirements^3–5^. The ability of the grating interferometer to operate at relatively low coherence requirements overcame these restrictions. Studies of vortex structures, magnetic domains and domain wall kinetics in the bulk of superconductors and ferromagnets which were not amenable to any other technique have been reported^6–11^. In addition, the high sensitivity of the apparatus was found to qualify it an excellent tool for the investigation of micro-structural features based on coherent scattering length density variations in bulk materials and objects^12–15^. In contrast to conventional instrumentation, the combination of microstructural sensitivity with macroscopic real space resolution enables the study of heterogeneous structures and processes in representative volumes^16–18^. Due to analogies to dark-field microscopy, this phase imaging technique is in neutron imaging referred to as dark-field contrast imaging^12^.

Today grating interferometers for imaging are available at many leading instruments at neutron sources around the world^1,12,19–22^. Numerous successful studies^6–18^ triggered continuous developments with regards to varying implementations of analogue techniques^7,21–26^. Key progress was established in particular by the quantitative interpretation of dark-field contrast providing correlation length information^14,15^. The first quantitative characterization of microstructures was reported on the nano-scale already when a spin-echo interferometer was successfully introduced to neutron imaging^24^. It operates in full analogy to Talbot Lau grating interferometric imaging but builds on the interference of the two spin states of the neutron wave function. The advantage of this method is the access to the nano-scale, full remote control of the modulation as well as its suitability for the most efficient time-of-flight wavelength dispersive measurement approach with neutrons at pulsed spallation sources^24,25^. Disadvantages are the sophisticated and elaborate set-up and technological barriers to assess larger length scales towards the micro-scale, without excessive sample to detector distances. The micrometer scale is however covered by the common Talbot Lau interferometry^15–18^. A more recent implementation, referred to as far-field multi-phase-grating interferometer^26^, implies similar issues towards the conventional range, but in addition faces efficiency issues due to a high general collimation requirement.

Here we demonstrate that the interferometric nature of the applied techniques is not relevant but only the spatial modulation of the beam, no matter how it is achieved. In contrast to previous works we chose the most basic approach to create spatial beam modulation on a suitable length scale to retrievably encode small angle beam deviations superimposed to real space images^14^. We prove that we are thus able to record and analyse the corresponding multi-modal images providing attenuation contrast, differential phase and dark-field contrast in full analogy to the discussed interferometric techniques. Similar approaches of non-interferometric phase imaging through structured illumination are known in light microscopy^27^ and x-ray imaging^28^.

## The Single Grating Method

**Figure 1:**
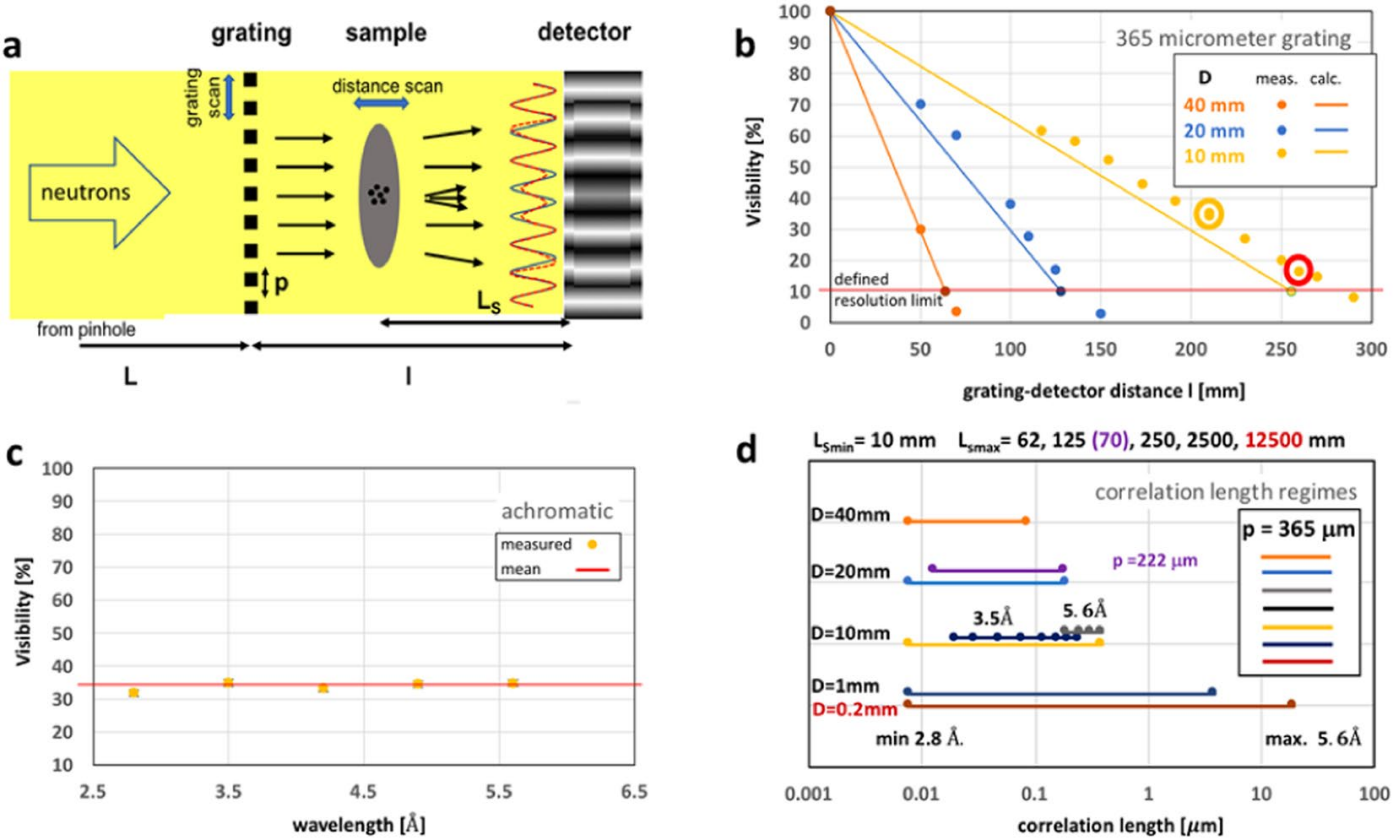
Set-up and parameters. (**a**) Sketch of the basic set-up. The principles of differential phase and scattering detection (dark-field contrast) are depicted: blue the undisturbed, red the refracted and scattered pattern. (**b**) Visibilities V achieved for L = 7 m pinhole to grating distance for different standard pinhole sizes D. Lines are based on the calculated geometric blur d = l/(L/D). A red circle indicates the parameters used for the presented study. (**c**) Measured proof of achromatic visibility with data taken at the point marked with yellow circle in (**b**). (**d**) Achievable correlation lengths according to Eq. (2)^14^ for standard pinhole sizes available at the benchmark instrument ICON at PSI^29^ and D = 0.2 mm like utilized for far-field interferometry^26^. Minima of indicated ranges refer to L_S_ = 10 mm and a minimum wavelength of 2.8 Å, maxima to L_S_ according to (**b**) and a wavelength of 5.6 Å. At D = 20 mm a comparison with the use of a 222 μm period grating is provided and at D = 10 mm the parameters utilized in the presented study are shown.

The set-up is established by merely adding an attenuation grating in a conventional pinhole collimated imaging instrument (Fig. 1). The image of the grating will be an accordingly spatially modulated intensity profile. Due to the geometric blur the modulation image will feature a sinusoidal intensity distribution, comparable to these of interferometric methods. The achievable visibility V = (I_max_ − I_min_)/(I_max_ + I_min_) depends, for a perfect absorption grating, only on the resolution capability of the set-up and is fully independent of the wavelength, hence fully achromatic in contrast to any approach presented earlier (Fig. 1b,c). This modulation of the beam which is simply a projection image of an absorption grating will be used in analogy to all interference based beam modulations before to provide quantifiable spatially resolved measurements of attenuation, phase and scattered wave interference. The principle of the latter is schematically depicted in Fig. 1a.

The sensitivity of a modulated beam measurement technique depends on the modulation period *p* and the sample to detector distance *L*_*s*_ (Fig. 1a) and can in the small angle approximation be expressed by the characteristic angle or the probed scattering vector length

1 $${\theta }_{c}=p/2L{s}_{s}\,{\rm{and}}\,{q}_{c}=\pi p/\lambda {L}_{s},$$

respectively. At this angle, or scattering vector, intensity is shifted from the maxima exactly into the intensity depleted minima – the dark-field - of the modulation pattern. This in turn for the probed length scale equals^14^

2 $$\xi =\lambda {L}_{s}/p.$$

For the intrinsically achromatic technique the wavelength λ is a free parameter (Fig. 1c), but it is limited by the typically used spectra in the thermal and cold energy range. Both other parameters, *L*_*s*_ and *p*, are limited by the spatial resolution ability of the set-up (Fig. 1b). A larger distance and a smaller period provide access to larger structures (Fig. 1d). A typical Talbot Lau interferometer provides modulation conditions enabling to resolve micrometer sized structures with micrometer modulation periods. These require analyser gratings to be resolved. Here we consider periods of some 100 micrometer which can be resolved spatially up to some 100 mm distances by the detector directly. A workable compromise between visibility and resolvable range has to be found (Fig. 1b,d). Theoretical approximations as well as measurements confirm the ability to create modulations with substantial visibility and compatible with outstanding correlation length ranges probed from the nanometer to micrometer scale with standard instrument settings (Fig. 1b,d). This applies despite the limited wavelength range of 2.8 to 5.6 Å accessible with the utilized instrumentation. Figure 1d, in addition, displays the unparalleled extensive range our technique covers when employing slits as reported to be applied for far field interferometry^26^. For our demonstration an intermediate regime with the potential to cover nearly two orders of magnitude in correlation lengths has been chosen while analyzed data are restricted to correlation lengths ranging from about 10 to 300 nm.

## Measurements and Results

The measurements were performed with a 10 mm pinhole, common for conventional high resolution imaging measurements and leading to a collimation ratio *L/D* of about 700 at the ICON imaging instrument^29^. The chosen *L/D* and grating to detector distance *l* of 0.26 m enabled a visibility of 18% (Fig. 1b) comparable to such utilized in neutron Talbot Lau interferometry. The grating consisted of 20 μm high Gd lines on a quartz wafer with a duty cycle of 50% and a period of 365  μm. The detector used was a common combination of a 20 μm thick gadolinium oxysulfide scintillator screen and a CCD camera (Andor, iKon-L) with a 100 mm Zeiss photographic lens system. The corresponding field of view (FoV) was 70 × 70 mm^2^ with an effective pixel size of 35 μm and an intrinsic spatial resolution of around 70 μm. Note, that the pixel size was about a factor of ten smaller than the grating period, providing a sufficient number of measurement points to well resolve the modulation. The grating had an effective size of 50 × 50 mm^2^. The exposure time per image was 3 times 120 sec irrespective of the wavelength used.

A macroscopic beam modulation superimposed to an image enables different routes of measurement and analyses strategies for the contrast modalities^12,24,28,30^. While in principle it is possible to fully analyse such images for all modalities from a single shot^28,30^, here, again in analogy to Talbot Lau interferometry, a grating scan has been performed with 11 steps over one period. This enables conventional pixel-wise extraction of all three contrast parameters: transmission *A*, differential phase parameter *ϕ* and visibility *V* = *B/A* constituting the measured image as

3 $${I}_{i,j}={A}_{i,j}+{B}_{i,j}sin({C}_{i,j}X+{\phi }_{i,j})$$

where i, j are the pixel indices, C is the phase of the open beam modulation and X is the grating scan parameter.

**Figure 2:**
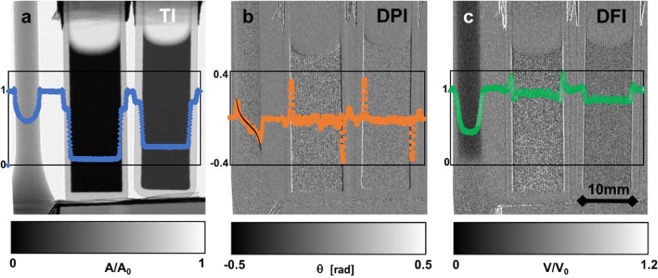
Three imaging modalities. Are measured simultaneously^12^; the images display a measurement of three samples, a 304 L steel cylinder and two quartz glass cuvettes containing aqueous solutions; (**a**) the conventional transmission image (TI) A/A_0_ (Eq. 3); (**b**) the differential phase image (DPI) *ϕ* (Eq. 3), the line profile contains a theoretical calculation for the steel cylinder (black curve)(Eq. 4); (**c**) the dark-field image (DFI) reflects the relative visibility loss through scattering from the sample V/V_0_ = BA_0_/(B_0_A) (Eq. 3).

For an individual measurement of a set of samples the images corresponding to the three parameters *A* (TI), *ϕ* (DPI) and *V = B/A* (DFI) are depicted in Fig. 2. The samples in this measurement are a cylindric tensile test sample of 304 L steel and two aqueous solutions in quartz glass cuvettes. All three contrast modalities display the typical corresponding features with respect to the samples, which are well-known from corresponding interferometric imaging approaches. Due to the good spatial resolution capability of the set-up and the significant sample to detector distance even the signature of far field phase contrast^3^ is visible upon careful inspection in the attenuation contrast image (TI, Fig. 2a). The differential phase contrast image depicts the influence of the distorted neutron wave-front on the spatial modulation phase *ϕ* (Fig. 1a). The respective local neutron wave phase shift can be extracted from this according to the relation^1^

4 $$\phi =\frac{\lambda {L}_{S}}{p}\frac{\partial \Theta }{\partial x}$$

where ∂Θ/∂x is the gradient of the neutron wave-front perpendicular to the modulation. The phase can be retrieved straightforwardly from corresponding integration^1^. For the phase profile of the cylindric steel sample we compare the measurement to the according calculation of the differential phase in Fig. 2b, underlining good quantitative agreement within the spatial resolution limit.

Systematic quantitative reference measurements have been performed on a second set of samples. The study focusses on quantitative dark-field imaging^12,14–18^. The first reference samples were two commercially available fractal powders, namely Sipernat-350 and Sipernat-310 with characteristic particle sizes of 4 and 8.5 micrometers, respectively^31^. Sipernat-310 is a powder of silica featuring a large surface area of 700 m^2^/g. Together with the micrometer sized particle characteristics this makes Sipernat-310 a well-suited material to investigate basic characteristics of cohesive powders. It has recently been used in a study observing with spatial resolution the heterogeneous breakdown of the fractal microstructure^18^. This study served as reference.

Both powders were measured contained in quartz glass cuvettes with sample thicknesses of 5 mm for both Sipernat-310 and −350 and additionally a 2 mm thick sample of Sipernat-350. Distance scans were performed at wavelengths of 3.5 Å and 5.6 Å from L_s_ 20 to 250 mm and 120 to 250 mm, respectively (compare Fig. 1b). The measured visibilities from grating scans at each of these settings were analysed pixel-wise using our standard Talbot Lau data reduction software^32^. The normalized visibilities can be written as^14^

5 $$\frac{V(\xi )}{{V}_{0}}={e}^{\sum t(G(\xi )-1)}$$

where *V*_0_ is the visibility without sample, *Σ* is the total small angle scattering cross section and *G(ξ*) is the projected real space correlation function of the microscopic sample structures^14^. The data is further reduced pixel-wise by computing the logarithm and dividing by the sample thickness *t* and the wavelength square. The total scattering probability Σ*t* depends on *λ*^2^, which needs to be normalized in order to combine scans at different wavelengths and to achieve results independent of the utilized beam. Subsequently the data is modelled with specific projected real space correlation functions *G(ξ)* representative of the microscopic structure investigated. A fitting procedure finally enables to retrieve the respective structural parameters.

**Figure 3:**
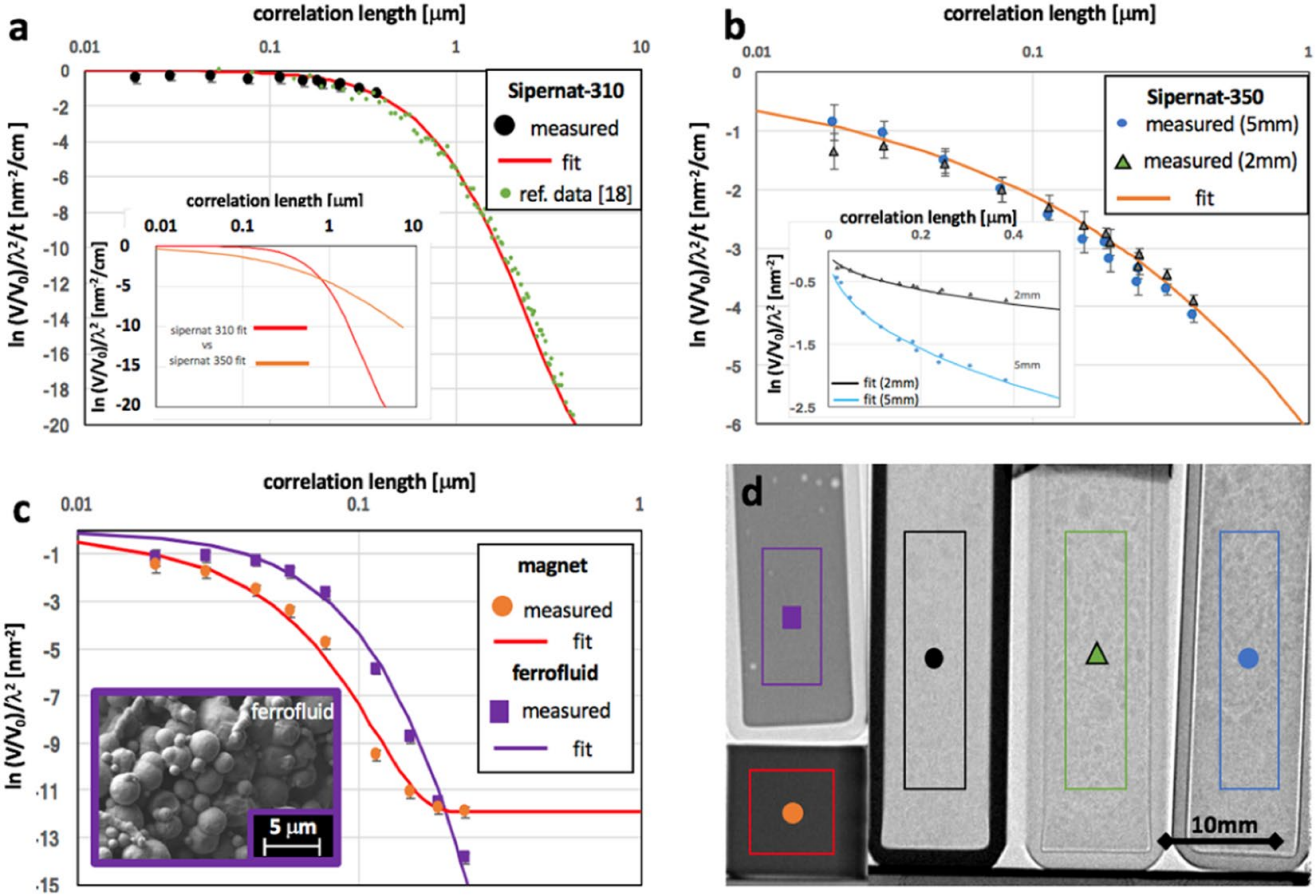
Mapping scattering length density correlations. Quantitative dark-field imaging performed through variation of wavelength and sample to detector distance of samples as shown in the TI in (d); (**a**) results of measurement on a silica powder (Sipernat-310^31^) combined with SESANS data and a model fit from literature;^18^ the inset compares the applied model to the model used for the related powder sample presented in (**b**). Results of Sipernat-350^31^ powder with model fit (red line); insert displays the model applied separately to the results for the two different sample thicknesses before normalization. (**c**) Data and fit for ferrofluid sample (violet squares) and a porous solid ferromagnet cube (red circles) and corresponding model fits. (**d**) An attenuation contrast TI indicating samples with symbols and colors used in the corresponding plots.

According to ref. ^18^. Sipernat-310 is best modelled with a randomly distributed two-phase medium^33^ corresponding to

6 $$G(\xi )=\frac{\xi }{a}{K}_{1}(\frac{\xi }{a})$$

where *K*_1_ is the modified Bessel function of second kind and first order and a represents the characteristic structure size in the two-phase medium. The model and parameters found through small angle scattering measurements in ref. ^18^, where a = 1.56 μm and Σ = 0.45, fit the single grating data as depicted in Fig. 3a. This model, however, did not fit the data of Sipernat-350, the second powder sample (compare inset Fig. 3a). This correlates with the fact that the powders are significantly different in their specific structure and surface area, which for Sipernat-350 is 55 m^2^/g, compared to 700 m^2^/g of Sipernat-310^31^. The two data sets from two different sample thicknesses of Sipernat-350, Fig. 3b inset, could both be fitted with the same model

7 $$G(\xi )={e}^{[-{(\xi /a)}^{\alpha }]}.$$

This model describes a simplified limited fractal taking into account an exponential cut-off ^34^. The two data sets after normalization with their respective thicknesses coincide well (Fig. 3b). The fit yields a characteristic length scale a = 7 μm and α = 0.5, with α being related to the structure of the phase boundary. Here α ranges in the lower domain of 0 <α < 1 which corresponds to open and branched distributions with high specific surfaces.

Additional data on well characterized samples returned the porosity on the probed length scale of a ferrite magnet produced through sintering as well as the poly-disperse structure size distribution in a ferrofluid (Fig. 3c). The cubic ferrite sample of 10 mm side length consisting of Fe_2_O_3_ (86%) and SrCO_3_ (14%) is revealed to have significant porosity in the probed size range constituting about 1% of the volume. The pores are characterized with a poly-disperse size distribution with predominant sizes of 20 and 210 nm diameter. Hard sphere models^14,34^ were used to approximate the pore structure and remaining deviations can be assumed to be due to a deviation of the average pore shape from the spherical model. The volume fraction of the smaller pores is found to be more than a factor two lower than the one of the dominating 200 nm pores constituting a volume fraction of 0.6%. In addition, microscopy reveals, that pores can also be found on the scale of several micrometers, which is, however, beyond the probed length scale and does not influence the presented results. The ferrofluid can be described by a random two-phase medium (Eq. 6) consisting of a polydisperse particle distribution (inset Fig. 3c) suspended in a liquid. Analyses provides a characteristic size parameter of a = 250 nm, corresponding to the smallest particles found in the liquid. In addition, the largest length scale derived from model fitting coincides with the largest particle dimension in the ferrofluid of about 3 μm (Fig. 3c). The deviations of the measured points from the fit at the lowest probed correlation lengths are understood to be due to incoherent scattering from the hydrogenous liquid phase.

## Conclusions

We conclude, that our most simple approach of a single attenuation grating with sub-millimeter period that requires no specific and sensitive alignment, does overcome stability requirements, design and fabrication issues related to fine structures as well as other limitations of interferometers. In particular the intrinsically achromatic nature of our technique enables straightforwardly multi-wavelength and polychromatic studies without the corresponding drawbacks of interferometry. In contrast, it provides the capability for efficient, simple, flexible and quantitative multi-modal imaging and especially phase imaging in the form of differential phase and dark-field contrast. In addition, the method holds the potential to straightforwardly cover an unprecedented correlation length range. The capability to extend the range reported for Talbot Lau interferometers^14–18^ by more than an order of magnitude into the nanometer range has been demonstrated. Flux densities available even at medium flux sources allow to further increase collimation ratios within standard imaging settings (Fig. 1). This enables to substantially increase the total covered correlation length scale range from the nanometer regime into and beyond the typically assessed micrometer range of neutron Talbot Lau interferometry. In particular a comparison with recent approaches of far-field interferometers^26^ (compare Fig. 1d) suggests that similar collimation conditions lead to significantly superior performance parameters. This implies that our much simpler and in contrast non-interferometric and truly achromatic set-up can provide higher efficiency with at least the same accuracy and range of experiments without the need for careful alignment, stability and interferometry per se. Thus, the introduced approach constitutes a paradigm shift for corresponding measurements and methods.

Note, that in principle the technique does not require a pinhole, but could use a slit or even a grating in order to increase the flux on the sample. The downsides are, however, like in other far field methods and for extended sample distance scans in other set-ups, that real space resolution of the sample is lost accordingly and that in the latter case furthermore an additional grating adds complexity again. On the other hand, the single grating could be moved towards the detector in the sample distance scan, together with the sample, and the pinhole collimation could be relaxed correspondingly, providing an additional efficiency gain.

Furthermore, especially the wider spectral range accessible continuously at high flux spallation neutron sources generates substantial benefit from the achromatic nature of the set-up for large range high flux measurements including kinetic studies. Instruments like the imaging beamline ODIN under construction at the European Spallation Source^35^ cover wavelength ranges of up to one order of magnitude. This implies that a correlation length range of the same order of magnitude can be probed simultaneously, enabling the spatially resolved observation of microstructural changes not only in time-dependent but non-uniform environments such as e.g. in shear fields, flow, inhomogeneous temperature, pressure and magnetic fields in full field observations. This paves the way to foster entirely new analytical capabilities for complex inhomogeneous and non-equilibrium structural states in materials either opaque to or not providing sufficient contrast with other types of radiation.

An extension to 2-dimensional phase contrast sensitivity and resolution is similarly straight forward through the utilization of 2D absorption patterns in contrast to recent elaborate approaches with interferometry^23^. This enables to additionally study micro-structural anisotropies without the need of several sample scans.
